# The Paradoxical Effect of Inclusive Leadership on Subordinates’ Creativity

**DOI:** 10.3389/fpsyg.2019.02960

**Published:** 2020-01-22

**Authors:** Jinqiang Zhu, Shiyong Xu, Bainan Zhang

**Affiliations:** ^1^School of Management, Minzu University of China, Beijing, China; ^2^Center for Human Resource Development and Assessment, School of Labor and Human Resources, Renmin University of China, Beijing, China; ^3^School of Labor and Human Resources, Renmin University of China, Beijing, China

**Keywords:** inclusive leadership, psychological safety, challenge-related stress, creativity, paradoxical effect

## Abstract

Previous research about inclusive leadership and creativity has produced contradictory results. The present study tried to explain the contradictory findings based on the antecedent-benefit-cost framework (ABC). We found that inclusive leadership promoted subordinates’ creativity by enhancing subordinates’ psychological safety but discouraged subordinates’ creativity by reducing challenge-related stress. The present study illustrated the complex mediating mechanism of inclusive leadership’s impact on creativity, presenting a complementary explanation of the conflicting relationships between inclusive leadership and creativity. In addition, we validated the ABC framework.

## Introduction

Employee creativity has been deemed to be one of the most key factors driving business success (Suk et al., 2015). A significant number of scholars and practitioners have placed attention on the influence factors of creativity to develop subordinates’ creativity (Amabile and Herron, 1996). Previous research found that leaders play a key role in developing subordinates’ creativity (e.g., Suk et al., 2015; Randel et al., 2018), therefore, researches about the effect of leadership on subordinates’ creativity have been arousing much concern among scholars (e.g., Zhang and Bartol, 2010; Liu et al., 2012).

Researchers and practitioners have increasingly looked to inclusive leadership, which is defined as “*leaders exhibit openness, accessibility, and availability in their interactions with followers*” (Carmeli et al., 2010), as a route to increased levels of creativity (Carmeli et al., 2010; Randel et al., 2018) to reconcile diverse individuals in the workplace. The focus of prior researches about the effect of inclusive leadership on creativity has predominantly been centered on its positive role (Carmeli et al., 2010). For example, Ye et al. (2019) found that inclusive leadership increased team innovation. Studies by Wang et al. (2019) and Fang et al. (2019) showed that inclusive leadership was positively related to innovation. Frost (2018) found that the combination of inclusive leadership and diverse teams can promote innovation. However, as is known to all in China, “a kind mother makes a wastrel,” some scholars have begun to argue that inclusive leadership also has the negative impact on employees’ behavior (Gu et al., 2017), which have been proved by some recent empirical studies (Zheng et al., 2018).

Scholars tried to explain those contradictory results from the view of context (Zhang et al., 2016; Cai et al., 2017). They found moderation variables that impact the relationship between inclusive leadership and creativity (Zhang et al., 2016). For example, Zhang et al. (2016) argued that the positive effects of perceived inclusive leadership by subordinates were weaker for those subordinates within high power distance culture where keeping distance between subordinates and superiors is required, thus hindering the development of benign relationship between them, and then weakening the effectiveness of inclusive leadership. Ye et al. (2018) got the same conclusion.

However, more and more scholars argued that the effects of inclusive leadership on creativity possess complex mediating process (Gu et al., 2017). Randel et al. (2018) argued inclusive leadership affects subordinates’ behavior through affecting subordinates’ cognition. Suk et al. (2015) contended that it is very important to empirically test the possible cognitive mechanisms between inclusive leadership and subordinates’ creativity. Thus, for achieving effective use of inclusive leadership to impact creativity, one important aspect to address is to find the role of related cognitive mechanisms.

**FIGURE 1:**
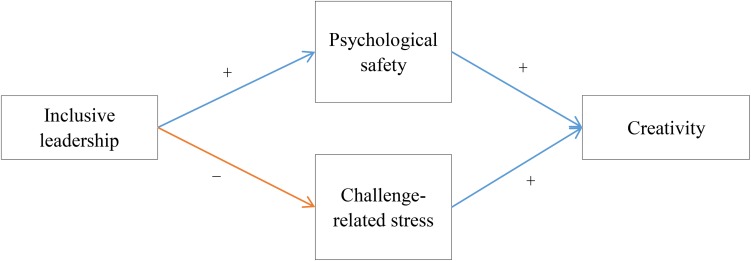
Theoretical model.

Most existing research mainly focused on single cognitive mechanism, especially emphasized the positive mechanisms via which inclusive leadership positively affecting creativity (Carmeli et al., 2010). Recently, a significant theory which is called antecedent-benefit-cost framework (ABC) has been developed by Busse et al. (2016), this theory argued that an antecedent variable has a paradoxical effect on the dependent variable through two different mediation variables. Accordingly, the term *benefits* are used to denote desirable immediate outcomes and the term *costs* to denote any undesirable immediate outcomes. Drawing on the ABC theory, we argue that inclusive leadership has a paradoxical effect on creativity through two different mediation variables. Given this viewpoint, the present study aims to identify the underlying mediation mechanisms of inclusive leadership impacting creativity in opposite directions. By integrating ABC framework and previous studies, we argue that inclusive leadership promotes subordinates’ creativity through cognitive mechanisms (i.e., the psychological safety), but discourages subordinates’ creativity through motivating mechanisms (i.e., the challenge-related stress). The theoretical framework is presented in Figure 1.

Our work has the following contributions. First, our approach takes a more comprehensive perspective to understand the complex mediation mechanism of inclusive leadership’s effect on creativity. Prior work of inclusive leadership mainly examined beneficial mediator variables (Carmeli et al., 2010), but the cost mediator variables are overlooked. By integrating ABC framework and existing literature, we explore the benefit and cost mediation variables in one model and identify two specific cognitive and motivating mechanism of the ABC framework in the leadership field. Second, previous studies tried to explain conflicting findings of inclusive leadership and creativity from the view of context (e.g., Zhang et al., 2016). However, the shortcoming of these explains like Zhang et al. (2016) is that the impacts of inclusive leadership on creativity are constrained to the specific context and thus lack generalizability. We introduce the ABC framework to leadership field by presenting a complementary explanation of the contradictory results about inclusive leadership and creativity from the view of the mediation process. Finally, our study enriches the literature about inclusive leadership. It is very important to understand that inclusive leadership’s “bright side” and “dark side” may coexist. Our study will explore the paradoxical effects of inclusive leadership on subordinates’ creativity.

## Theoretical Background and Hypotheses

### Inclusive Leadership and Creativity

Carmeli et al. (2010) defined inclusive leadership as leaders exhibit openness, accessibility, and availability in their interactions with followers. Randel et al. (2018) systematically compared inclusive leadership with other existing leadership styles such as empowering leadership, transformational leadership, authentic leadership, servant leadership, and leader-member exchange. The most notable differences between inclusive leadership and other leadership styles are that the former emphasizes on subordinates’ perceptions of belongingness and their diverse contributions (Randel et al., 2018).

Creativity refers to the production of novel, useful, and value ideas (Zhou and George, 2001). Inclusive leadership focuses on fostering group members’ perceptions of both belonging and value for uniqueness as a group member (Randel et al., 2018), which are important influence factors of creativity (Scott and Bruce, 1994). Many empirical studies showed that inclusive leadership significantly impact creativity (Carmeli et al., 2010; Choi et al., 2015; Javed et al., 2017).

The ABC theory points out that two competitive mediations lead to the antecedent variable which could impact the dependent variable in opposite directions. A positive indirect effect via a benefit variable B and a negative indirect effect via a cost variable C. The psychological safety and challenge-related stress has been found to be reliable and universally underlying cognitive processes that links leadership style to employees’ behavior in the leadership literature (Carmeli et al., 2010; Montani et al., 2017; Zhu and Zhang, 2019). Drawing on ABC theory and related studies, we propose that inclusive leadership has a paradoxical effect on creativity through psychological safety and challenge-related stress.

### The Cognitive Mechanism: The Mediating Effect of Psychological Safety

Kahn (1990) defined psychological safety as “*the subjective perception that employees feel to have ability of showing their selves without fear of negative consequences for self-image, status, or career*.” Based upon the ABC theory, we propose that inclusive leadership promotes subordinates’ creativity through enhancing subordinates’ psychological safety. The inclusive leader is characterized as fostering subordinates’ perceptions of belonging (Randel et al., 2018), which help to develop psychological safety. Inclusive leaders are approachable, caring subordinates and helping subordinates, which helps to build high-quality leader-member relation (Carmeli et al., 2010) and making subordinates develop psychological safety (Carmeli and Gittell, 2009). Inclusive leaders encourage subordinates to propose new ideas, listen to their opinions, be willing to communicate with subordinates, be willing to discuss problems and solutions with their subordinates, and tolerate subordinates’ mistakes and failures (Wasserman et al., 2008; Carmeli et al., 2010). That will make subordinates think that innovative ideas are recognized, are encouraged by leaders, and they (the subordinates) are not punished for failure, thus subordinates will develop psychological safety. Existing empirical studies showed that managerial openness and benign supervisor-subordinates relation improve subordinates’ psychological safety (Li et al., 2015).

Creativity refers to the production of novel, useful, and value ideas (Zhou and George, 2001). Novelty, which often introduces uncertainty, is at the center of the definition (Carmeli et al., 2010). Creativity tends to be a risky endeavor (Zhu and Zhang, 2019), therefore, subordinates will avoid creativity in that they are afraid to be punished for failing creativity. Subordinates will evaluate the possible negative consequences of failure in creativity before they engage in creativity. Thus, signals for safety are one of the most important factors related to creativity (Carmeli et al., 2010). West and Richter (2008) found that when feeling psychologically unsafe, employees are less likely to display creativity at work. Psychological safety can improve subordinates’ ability to focus (Li and Yan, 2007), then prompting employees to find new solutions (Carmeli et al., 2014).

Taking all these into consideration, inclusive leadership makes subordinates feel psychologically safe to perform creative activities, making subordinates more likely to engage in creative activities. Past empirical studies proved that psychological safety is an important cognitive process which links leadership and subordinates’ behavior (Hirak et al., 2012; Zhu and Zhang, 2019). For example, the study by Carmeli et al. (2014) showed that transformational leadership improved subordinates’ creative problem-solving through psychological safety. Hence, we propose the following hypothesis:

Hypothesis 1: Inclusive leadership has a positive impact on creativity through enhancing subordinates’ psychological safety.

### The Motivating Mechanism: The Mediating Effect of Challenge-Related Stress

Challenge-Related stress is defined as a kind of stress related to positive work outcomes and creates challenge and feelings of fulfilment or achievement. Examples of the challenge-related stress include job overload, high levels of responsibility, and time pressures (Cavanaugh et al., 2000). In the workplace, leadership behavior is an important event that causes employees stress and is an important source of stress for employees (Li et al., 2012). Inclusive leaders care about and meet subordinates’ needs (Carmeli et al., 2010). When subordinates have not enough time to complete a job, inclusive leaders will give them more time, which will reduce time pressures.

Inclusive leadership was characterized as having the abilities to tolerating subordinates’ mistakes and failures (Wasserman et al., 2008). If the leader blindly accepts the mistakes and failures of subordinates, it will lead subordinates to make mistakes without worrying (Zhu et al., 2018) because they may believe that there is no punishment. Therefore, employees may care little about their work performance, which reduces the sense of responsibility of employees for their work. After employees have made mistakes, the inclusive leader does not punish employees, resulting in employees coming to a standstill and becoming fat cats (Zhu et al., 2018). All will make subordinates become unambitious and trapped in the muddle along mentality, such problem might be more common in those countries with highly centralized culture where inclusive leadership will rob subordinates’ urgency and ambition. Time pressures and high levels of responsibility belong to challenge-related stress (Cavanaugh et al., 2000). We therefore theorize that inclusive leadership will reduce subordinates’ challenge-related stress.

The challenge-related stress, such as job overload, high levels of responsibility, and time pressures, make subordinates feel that they are valued by leaders (Qing and Zhang, 2014) because subordinates may perceive that leaders are convinced of subordinates’ ability to complete more job in less time and have high expectation to subordinates. This will activate subordinates’ intrinsic motivation (Qing and Zhang, 2014), which is one important influence factor of creativity. When an individual has fewer motivational resources to perform a certain behavior, the individual will reduce such behavior (Bandura, 2001). The challenge-related stress, as a kind of important motivational resources, can motivate subordinates to innovate (Zhang, 2015). When the challenge-related stress of subordinates reduce, subordinates will have no sufficient motivation to innovate (Liu et al., 2010). Existing empirical studies showed that challenge-related stress had a significantly positive influence on employees’ creativity (Qing and Zhang, 2014; Zhang, 2015; Montani et al., 2017). Hence, we propose the following hypothesis:

Hypothesis 2: Inclusive leadership has a negative effect on creativity through reducing subordinates’ challenge-related stress.

## Materials and Methods

### Sample and Procedure

Full-time and front-line employees of two organizations [one is an information technology organization (IT) and the other is a research organization] in mainland China participated in this study. These two companies were famous and in the list of 500 largest companies in China. Employees participated the study were programmers in the IT company and researchers in the research organization. After giving informed consent, links to the online survey were sent to participants. To avoid the potential problems associated with common method bias, we collected data in two waves. Participants rated inclusive leadership and provided their demographics at wave 1 and, 1 month later, rated their psychological safety, challenge-related stress and creativity at wave 2. We used participants’ email to match the two-wave data. The time of participants answering questionnaires was used to identify whether participants randomly answer the online questionnaires.

We obtained the list of employees from the two companies with the help of HR managers. We asked the HR magagers to find out which employees would like to participate in our study and then provide us the employees’ name and email. Then one of authors sent the links to employees. A total of 393 individuals answered the wave 1 questionnaires and 289 individuals answered the wave 2 questionnaires. 45 questionnaires were dropped because of the short answering time and mismatch between the wave 1 and wave 2. Our final sample included a total of 244 observations with complete data across the wave 1 and wave 2 surveys. Results of *T*-tests showed no significant differences on gender (M_responders_ = 1.56, M_nonresponders_ = 1.56, *T* = −0.044, *p* > 0.05), age (M_responders_ = 31.70, M_nonresponders_ = 31.55, *T* = 0.226, *p* > 0.05), marry (M_responders_ = 1.66, M_nonresponders_ = 1.61, *T* = 0.983, *p* > 0.05), tenure (M_*responders*_ = 9.01, M_*nonresponders*_ = 9.88, *T* = −1.201, *p* > 0.05), education (M_responders_ = 3.80, M_nonresponders_ = 3.66, *T* = 1.717, *p* > 0.05), position (M_responders_ = 1.42, M_nonresponders_ = 1.44, *T* = −0.38, *p* > 0.05) and inclusive leadership (M_responders_ = 4.50, M_nonresponders_ = 4.39, *T* = 0.987, *p* > 0.05) existed between the wave 2 responders and non-responders (Dooley and Lindner, 2003). 66.00% of participants were married, 56.10% were male, 61.30% were ordinary employees, and 53.70% had a bachelor’s degree or above. The mean of participants’ age was 31.70 years (SD = 6.26) and the mean of tenure was 9.01 years (SD = 6.53).

### Measures

We conducted the study in China, and all scales have been validated in the Chinese context (Liang, 2014; Qing and Zhang, 2014; Zhu and Zhang, 2019).

#### Inclusive Leadership

We used Carmeli et al.’s (2010) nine-item scale (α = 0.94) to assess inclusive leadership. Subordinates’ perceptions of inclusive leadership (e.g., “My manager is open to hearing new ideas”) were measured on a scale (1 = strongly disagree, 6 = strongly agree).

#### Psychological Safety

Five items adopted from Li and Yan’s (2007) psychological safety scale were used to measure subordinates’ psychological safety (1 = strongly disagree, 6 = strongly agree). One reverse-worded led to low reliability. The value of the corrected item-total correlation (CITC) was −0.07 less than 0.4, thus we deleted the item (“I am afraid to express my opinions at work”). Four items exhibited high internal consistency (α = 0.83). An example of the items used was “I’m not afraid to be myself at work.”

#### Challenge-Related Stress

Six items (α = 0.93) were adopted from Cavanaugh et al.’s (2000) work stress scale. We asked respondents to rate how much stress each item (e.g., “The amount of responsibility I have”) causes them on a scale from 1 (no stress at all) to 5 (a great deal of stress).

#### Creativity

To measure creativity, six items adopted from Scott and Bruce’s (1994) creativity scale were employed (α = 0.94). Respondents were asked to indicate how often they engaged in each of the behaviors (e.g., “Generates creative ideas”) on a scale ranging from 1 (never) to 6 (always).

#### Control Variables

Prior research has shown that demographic variables may influence creativity (e.g., Zhang et al., 2014), thus we controlled for these variables like gender, age, marry, tenure, education, and position in our study.

### Data Analysis

The statistical software SPSS 22.0 and Mplus 7.0 were used to analyze data. First, confirmatory factor analyses (CFAs) were used to test the discriminant validity and the common method variance (CMV). Second, we used structural equation model (SEM) to examine the theoretical model (Anderson and Gerbing, 1988). Several goodness-of-fit indexes were used to evaluate the fit of the theoretical model (Hu and Bentler, 1999; Cheung and Rensvold, 2002). These fit indexes include the chi-square divided by the degrees of freedom (χ^2^/df), the Non-Normed Fit Index (NNFI), the Comparative Fit Index (CFI), and the Root Mean Square Error of Approximation (RMSEA). Third, we used the bias-corrected bootstrapping to examine the mediation because it possessed high power (Preacher and Hayes, 2008; Miočević et al., 2017).

## Results

### Discriminant Validity

We used a series of CFAs to test the discriminant validity of the variables. The CFAs results indicated that the theoretical four-factor model (inclusive leadership, challenge-related stress, psychological safety, and creativity) was a better fit to the data than other models (see Table 1). The average variance extracted (AVE) of inclusive leadership, psychological safety, challenge-related stress, and creativity were, respectively, 0.64, 0.55, 0.69, 0.73 greater than the critical value 0.5 and squared correlations between variables (Fornell and Larcker, 1981), indicating the discriminant validity of these variables is good.

**TABLE 1:** Results of confirmatory factor analyses.

	**χ^2^**	***df***	**χ^2^/*df***	**RMSEA**	**CFI**	**NNFI**	**Δχ^2^**	**Δ*df***
Model 1 (hypothesized four-factor model)	663.78	269	2.47	0.078	0.92	0.91		
Model 2 (psychological safety and challenge-related stress combined)	1030.40	272	3.79	0.107	0.84	0.82	366.62^∗∗∗^	3
Model 3 (inclusive leadership, psychological safety and challenge-related stress combined)	2012.33	274	7.34	0.161	0.63	0.59	1348.55^∗∗∗^	5
Model 4 (single-factor model)	3174.86	275	11.54	0.208	0.38	0.32	2511.08^∗∗∗^	6
Model 5 (unmeasured latent methods factor model)	643.82	264	2.44	0.077	0.92	0.91	–19.96^∗∗^	−5

**N* = 244, ^∗^*p* < 0.05, ^∗∗^*p* < 0.01, ^∗∗∗^*p* < 0.001.*

### Common Method Variance

Following Podsakoff et al. (2003), to assess CMV we conducted Harman’s single-factor test by exploratory factor analysis (EFA) and CFA. The results of EFA showed that the first factor only accounted for 29.98% of the total variance. The results of CFA showed that the single-factor model was a poorer fit to the data than the theoretical four-factor model, and the change in chi-square was significant [△χ2(6) = 2511.08, *p* < 0.001] (see Table 1), suggesting that CMV effect is not present in the current study.

Further, we conducted the unmeasured latent methods factor, that all items loaded on both this latent methods factor and trait factors (Podsakoff et al., 2003), to test CMV. A comparison of the latent methods factor model (χ^2^ = 643.82, *df* = 264, χ^2^/*df* = 2.44, RMSEA = 0.077, NNFI = 0.91, CFI = 0.92) and the theoretical model (χ^2^ = 663.78, *df* = 269, χ^2^/*df* = 2.47, RMSEA = 0.078, NNFI = 0.91, CFI = 0.92) indicated CFI no changing (Cheung and Rensvold, 2002). Thus CMV should not be a severe problem in our study.

### Descriptive Statistics

Table 2 shows the means, standard deviations and zero-order correlations between the key variables. As expected, inclusive leadership had a significantly positive correlation with psychological safety (*r* = 0.40, *p* < 0.001) and creativity (*r* = 0.24, *p* < 0.001). Inclusive leadership had a significantly negative correlation with challenge-related stress (*r* = −0.19, *p* < 0.01). Creativity had significant positive correlation with challenge-related stress (*r* = 0.16, *p* < 0.05) and psychological safety (*r* = 0.25, *p* < 0.001).

**TABLE 2:** Means, standard deviations, and correlations.

**Variables**	**Mean**	**SD**	**1**	**2**	**3**
1. Inclusive leadership	4.50	0.96			
2. Psychological safety	4.20	0.76	0.40^∗∗∗^		
3. Challenge-related stress	2.64	0.87	−0.19^∗∗^	−0.14^∗^	
4. Creativity	4.18	0.86	0.24^∗∗∗^	0.25^∗∗∗^	0.16^∗^

**N* = 244, ^∗^*p* < 0.05, ^∗∗^*p* < 0.01, ^∗∗∗^*p* < 0.001.*

### Hypotheses Testing

We conduct the SEM using Mplus to test the theoretical model. To assess the direct effect of inclusive leadership on creativity, we added the direct path from inclusive leadership to creativity. Results showed that both the alternative model (χ^2^ = 815.61, *df* = 396, χ^2^/*df* = 2.06, RMSEA = 0.066, NNFI = 0.90, CFI = 0.91) and the theoretical model (χ^2^ = 828.62, *df* = 397, χ^2^/*df* = 2.09, RMSEA = 0.067, NNFI = 0.90, CFI = 0.91) fit the data well. Following Cheung and Rensvold (2002), the CFI almost did not change when the direct path from inclusive leadership to creativity was included, suggesting that the theoretical model was the most preferred model in the current study.

**FIGURE 2:**
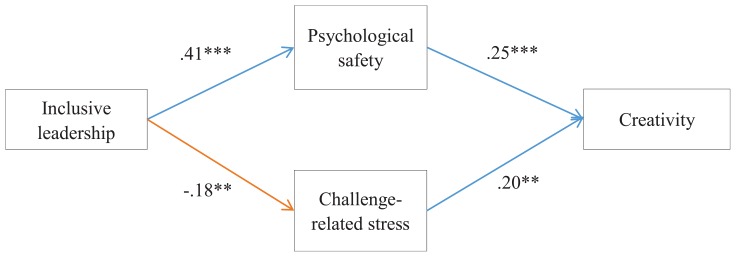
Results of theoretical model by using Mplus. *N* = 244, ^∗∗^*p* < 0.01, ^∗∗∗^*p* < 0.001. Standardized path coefficients are reported. Control variables are gender, age, marry, tenure, education, and position.

Figure 2 presents the results of SEM with the standardized coefficients. Hypothesis 1 describing the mediating effect of psychological safety was supported by the positive relationship between inclusive leadership and psychological safety (β = 0.41, *p* < 0.001) and the positive relationship between psychological safety and creativity (β = 0.25, *p* < 0.001). Furthermore, the indirect effect of psychological safety between inclusive leadership and creativity was significant (indirect effect = 0.10, Bootstrap = 5000, 95% CI = 0.014, 0.208, excluding zero). Together, these results support hypothesis 1.

Hypothesis 2 describing the mediating effect of challenge-related stress was supported by the negative relationship between inclusive leadership and challenge-related stress (β = −0.18, *p* < 0.05) and the positive relationship between psychological safety and creativity (β = 0.20, *p* < 0.01). Furthermore, the indirect effect of challenge-related stress between inclusive leadership and creativity was significant (indirect effect = −0.04, Bootstrap = 5000, 95% CI = −0.084, −0.010, excluding zero). Together, these results support hypothesis 2. The total effect was positive but not significant (total effect = 0.052, Bootstrap = 5000, 95% CI = −0.023, 0.138, including zero). The reason may be that the two opposing mediation effects offset one another.

## Discussion

Building on the ABC framework, the current study proposed and examined a dual mediation model of how inclusive leadership impacts creativity. Consistent with our predictions, inclusive leadership has a paradoxical effect on creativity through two different mediation variables. Specifically, inclusive leadership discouraged subordinates’ creativity through reducing challenge-related stress, but promoted subordinates’ creativity through enhancing psychological safety. Our work exposited the complex mediation process of inclusive leadership’s influence on creativity.

### Theoretical Implications

First, our findings extend the research of inclusive leadership. The positive effect of inclusive leadership has been frequently discussed in previous research (Carmeli et al., 2010; Randel et al., 2018), nevertheless, most of the existing studies did not capture or reflect the possible negative effect of inclusive leadership, especially through empirical research method (Randel et al., 2018). Our study empirically examined the negative effect of inclusive leadership, which bridged this gap and echoes the call from Randel et al. (2018) to uncover the “dark side” of inclusive leadership.

Second, our results suggest an alternative explanation for the negative relationship between inclusive leadership and creativity. Exiting literature tries to explain the negative relationship of inclusive leadership with creativity from the viewpoint of context (Zhang et al., 2016; Cai et al., 2017). However, to our knowledge, no existing studies ever tried to explore the negative impact of inclusive leadership’s mediating process on creativity. Drawing on the ABC framework, we empirically test the mediation effect of the challenge-related stress via which inclusive leadership negatively affects creativity, which presents an empirically supported complementary explanation.

Finally, our research found the paradoxical effect of inclusive leadership on creativity, which helps to resolve conflicting results from theoretical and empirical studies. Researchers found that inclusive leadership and creativity are sometimes positively and sometimes negatively related (Suk et al., 2015; Zhang et al., 2016; Gu et al., 2017), and tried to explain conflicting findings from the viewpoint of context (Zhang et al., 2016; Cai et al., 2017). However, the shortcoming of these explains like Zhang et al. (2016) is that the effects of inclusive leadership on creativity are limited to the specific context and thus lack generalizability. By introducing the ABC framework to the leadership field, we present a complementary explanation for the conflicting results observed to date regarding the relationship between inclusive leadership and creativity processes. Besides, by integrating ABC framework and related studies, we tested the benefit and cost mediation variables in one model to illustrate the underlying mechanisms of inclusive leadership having opposite directionalities impact on creativity. Most importantly, we identify two specific psychological mechanisms of the ABC framework in the leadership field, which verifies and extends the ABC framework.

### Practical Implications

The current study offers a few practical implications regarding to how to facilitate subordinates’ creativity by enhancing managers’ inclusive leadership style. To begin, it is very important to understand that the inclusive leadership’s “bright side” and “dark side” coexist. The organization should train managers to realize the “bright side” and “dark side” of inclusive leadership. Accordingly, managers should try their best to strengthen the “bright side” of inclusive leadership and reduce the “dark side” of inclusive leadership.

In addition, managers should pay attention to subordinates’ psychological safety and challenge-related stress. More importantly, when they want to promote subordinates’ creativity by facilitating subordinates’ psychological safety, managers should recognize that blind inclusion may reduce subordinates’ motivation. Thus, managers should take measure, such as activating subordinates’ challenge-related stress by building a competitive environment.

### Limitations and Future Research

First, the self-reporting nature of the data collection may be susceptible to CMV issues (Podsakoff et al., 2003). Although the two-waves design could help eliminate the concern for potential common method bias, it would be much better to use multi-sources data in future research. Specifically, subordinates’ creativity should be rated by their supervisor. In addition, given inclusive leadership may be also a team-level phenomenon, it is encouraged to develop a cross-level model to explore the cross effect of inclusive leadership on employees.

Second, although we recognize the culture’s pervasive influence, we did not consider culture as an important factor in this phenomenon. The study was conducted in China, which is characterized by the high power distance culture (Hofstede et al., 2010), referring to the extent to which inequality among persons in different positions of formal power is viewed as a natural (and even desirable) aspect of the social order (Brockner et al., 2001). Zhang et al. (2016) argued that culture of high power distance weakens the effectiveness of inclusive leadership because it hinders the development of a benign relationship between superiors and subordinates. Thus, culture should be considered as a potential variable in future researches. In addition, individual characteristics and team/organizational context factors may also influence the relationships which were tested in our paper. We encourage future research to examine those factors.

Finally, the present study only examined the cognitive mechanism and motivating mechanism through which inclusive leadership has opposite directional influences on creativity. We encourage future research to examine other underlying mechanisms, such as emotion mechanism, that may explain the possible “bright side” and “dark side” of inclusive leadership. In addition, the relationship between inclusive leadership and creativity may be non-linear. We encourage future studies to explore the curvilinear relationship between inclusive leadership and creativity.

## Data Availability Statement

The datasets generated for this study are available on request to the corresponding author.

## Ethics Statement

In this study, data were collected by JZ at the Minzu University of China. Institutions in China do not have Institutional Review Board. As a protection of all participants, all subjects read informed consent before participating this study and voluntarily made their decision to complete surveys. The protocol was approved by the Minzu University of China and funded as an important research project.

## Author Contributions

SX developed the theoretical model and hypotheses. JZ collected the data and wrote the manuscript. BZ provided comments on different versions of the manuscript and edited the manuscript.

## Conflict of Interest

The authors declare that the research was conducted in the absence of any commercial or financial relationships that could be construed as a potential conflict of interest.
